# Bacterial Species and Antibiotic Resistance—A Retrospective Analysis of Bacterial Cultures in a Pediatric Hospital

**DOI:** 10.3390/antibiotics12060966

**Published:** 2023-05-26

**Authors:** Bianca Prajescu, Liana Gavriliu, Mara Ioana Iesanu, Andreea Ioan, Anca Andreea Boboc, Catalin Boboc, Felicia Galos

**Affiliations:** 1 Department of Pediatrics, Marie Curie Emergency Children’s Hospital, 041451 Bucharest, Romania; bianca.dombici@rez.umfcd.ro (B.P.);; 2 Department for Prevention of Healthcare-Associated Infections, Marie Curie Emergency Children’s Hospital, 041451 Bucharest, Romania; liana.gavriliu@umfcd.ro; 3 Department of Infectious Disease, Carol Davila University of Medicine and Pharmacy, 020021 Bucharest, Romania; 4 Department of Physiology, Carol Davila University of Medicine and Pharmacy, 020021 Bucharest, Romania; 5 Department of Pediatrics, Carol Davila University of Medicine and Pharmacy, 020021 Bucharest, Romania

**Keywords:** antibiotic resistance, children, bacterial species, infection control measures, *Escherichia coli*, *Staphylococcus aureus*, *Klebsiella* spp., *Pseudomonas* spp., Gram-negative, Gram-positive

## Abstract

Antimicrobial resistance (AMR) has become a major healthcare concern having a rising incidence, especially in pediatric patients who are more susceptible to infections. The aim of our study was to analyze the bacterial species isolated from patients admitted to our tertiary hospital and their AMR profiles. We conducted a retrospective observational study by examining the bacterial cultures collected from pediatric patients admitted to our hospital over a period of one year. We identified the most common bacterial species from 1445 clinical isolates and their AMR patterns using standard microbiological techniques. Our analysis revealed that the most frequently isolated bacterial species were *Escherichia coli* (23.73%), *Staphylococcus aureus* (15.64%), *Klebsiella species* (12.04%), and *Pseudomonas species* (9.96%). Additionally, these species exhibited varying levels of resistance to commonly used antibiotics. Notably, we observed high rates of resistance among Gram-negative bacteria, including extended-spectrum beta-lactamase-producing *Escherichia coli* and *Klebsiella species*. Among Gram-positive bacteria, we observed a high level of methicillin-resistant *Staphylococcus aureus*. Our findings highlight the urgent need for effective antibiotic management programs and infection control measures to address the rising incidence of AMR in pediatric hospitals. Further research is needed to identify the mechanisms of resistance in these bacterial species and to develop new strategies for preventing and treating infections caused by antibiotic-resistant bacteria in pediatric patients.

## 1. Introduction

Antimicrobial resistance (AMR) is a major public health concern that affects individuals of all ages [1]. AMR is associated with high rates of mortality and increased medical costs, especially in low- and middle-income countries, due to the high burden of infectious diseases [2]. Pediatric patients are particularly vulnerable to antibiotic-resistant infections, as children are often exposed to antibiotics and have immature immune systems. Furthermore, bacteriemia is a leading cause of mortality among pediatric patients, and its treatment is threatened by the alarming increase in the prevalence of AMR [3]. Therefore, it is crucial to understand the prevalence and patterns of antibiotic resistance in pediatric hospital settings.

Global antibiotic consumption experienced a 65% increase worldwide between 2000 and 2015, showing an inverse correlation with the decline in deaths caused by infectious diseases. However, the extensive use of antibiotics has led to the emergence of antibiotic-resistant bacterial strains, complicating infection management and limiting the effectiveness of available antibiotics. Researchers predict millions of deaths due to bacterial AMR, highlighting the urgent need for action. To address this issue, implementing AMR surveillance systems is crucial to collect antimicrobial resistance data. This data can be used to develop empirical therapy and establish local and national antibiotic treatment guidelines. The World Health Organization (WHO) has introduced the ‘One Health’ concept, emphasizing the surveillance of AMR in humans, animals, and the environment through collaborative efforts between various research teams from different sectors [4].

The overuse and misuse of antibiotics, along with breaches in Infection Prevention and Control (IPC) and lack of policies and education, have led to the emergence of bacteria resistant to many commonly used antibiotics. AMR has several primary mechanisms, including the formation of biofilms, reduction of antibiotic permeability, and the use of active efflux pumps [5]. Additionally, novel AMR mechanisms are emerging despite efforts to control them. Multidrug-resistant pathogens (MDR) are of particular concern since they can cause infections that are challenging or impossible to treat with conventional antibiotic regimens. This, in turn, results in longer hospital stays, increased healthcare expenses, and higher mortality rates [6]. Some of the most frequent MDR bacteria includes extended-spectrum beta-lactamase producers (ESBL) *Escherichia coli* and *Klebsiella pneumonie*, carbapenem-resistant *Pseudomonas aeruginosa* (CRPA), carbapenem-resistant *Acinetobacter baumanii* (CRAB), carbapenem-resistant *Enterobacteriaceae* (CRE), methicillin-resistant *Staphylococcus aureus* (MRSA), vancomycin-resistant *Staphylococcus aureus* (VRSA) and vancomycin-resistant *enterococci* (VRE) [7].

There are various international surveillance networks, such as GLASS (Global Antimicrobial Resistance and Use Surveillance System), EARS-Net (European Antimicrobial Resistance Surveillance Network), CARSS (China Antimicrobial Resistance Surveillance System), and CHINET (China Antimicrobial Surveillance Network) [8,9]. However, these networks primarily focus on monitoring AMR in adults rather than addressing the issue in children. Furthermore, the prevalence distributions and AMR patterns of bacteria isolated from children differ significantly from those found in adults, as children cannot be considered mere “miniature adults” in the context of AMR [10,11]. Taking *Streptococcus pneumoniae* as an example, the rate of *S. pneumoniae* carriage is considerably higher in children (53%) compared to adults (4%), and the serotype distribution and antibiotic resistance patterns of *S. pneumoniae* also exhibit notable variations between the two age groups. For this reason, in 2015, China implemented the Infectious Diseases Surveillance of Pediatrics, including 11 tertiary care hospitals, but more international homogenous programs are needed [6].

The endorsed global action plan on AMR by the World Health Organization emphasizes the significance of promoting awareness of AMR through continuous monitoring and research initiatives across various regions worldwide. Monitoring AMR is crucial and offers several benefits, such as providing insights into bacterial resistance rates, facilitating the selection of suitable antibiotics, thereby reducing AMR rates, reducing hospitalization and treatment expenses, and lowering mortality rates [12,13,14].

In the present study, we aim to increase awareness of AMR among children by providing a description of the epidemiology and AMR rates of the main isolated bacterial pathogens in hospitalized patients between January 2022 and December 2022 at the tertiary center Marie Curie Emergency Children’s Hospital, Bucharest, Romania. The Marie Curie Emergency Children’s Hospital is a tertiary emergency hospital with 473 beds, catering to children from all over the country. The hospital houses several departments, including pediatrics, nephrology, cardiology, pediatric surgery and orthopedics, otolaryngology, cardiovascular surgery, neurosurgery, intensive care unit, and neonatal intensive care unit. 

## 2. Results

### 2.1. Distribution of the Main Isolates

There were 1445 clinical isolates, consecutive and non-duplicated, collected from the hospitalized patients, from which 932 were Gram-negative (GN) bacteria (64.49%), 503 were Gram-positive (GP) bacteria (34.80%), and 10 were fungi (0.69%). The most frequent ten isolated bacterial species were *Escherichia coli* (23.73%), *Staphylococcus aureus* (15.64%), *Klebsiella species* (spp.) (12.04%), *Pseudomonas* spp. (9.96%), coagulase-negative *Staphylococci* (8.85%), *Enterococcus* spp. (7.4%), other non-fermentative GN bacilli (4.42%), *Acinetobacter* spp. (3.52%), other Gram-negative bacteria (3.25%), and *Proteus* spp. (2.9%) (Table 1). Most commonly, the cultures were isolated from the urine, the respiratory tract, and the skin/nose/pharynx. The distribution of the specimens and the main five pathogens isolated from each are mentioned in Table 2, and the distribution per department is in Table 3.

### 2.2. Antimicrobial Resistance in Main Bacterial Species

#### 2.2.1. *Escherichia coli*

**Figure 1:**
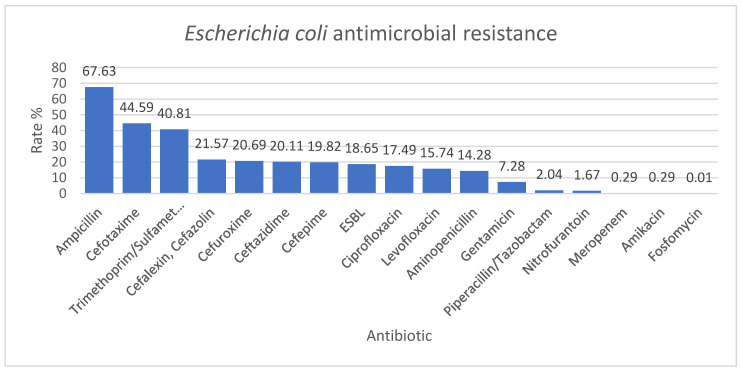
*Escherichia coli* antimicrobial resistance encountered in the specimens collected from the pediatric patients admitted to our hospital.

*Escherichia coli* was the most frequently isolated bacterium, with a proportion of 23.73%, and it was mainly isolated from urine (192/343), followed by stool (34/343) and peritoneal fluid (33/343) (Table 4). The highest resistance rate was identified to ampicillin (67.63%) and the lowest to amikacin, meropenem, and fosfomycin (0.29%, 0.29%, and 0.01%). ESBL *Escherichia coli* had a 49.42% rate (Figure 1).

#### 2.2.2. *Staphylococcus aureus*

**Figure 2:**
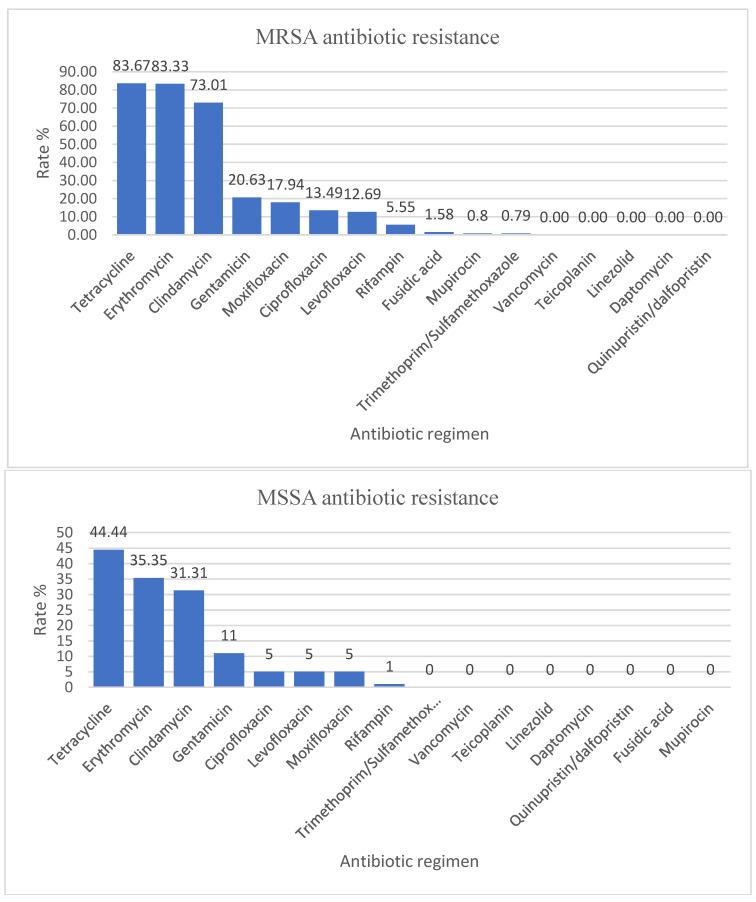
MRSA and MSSA antimicrobial resistance encountered in the specimens collected from the pediatric patients admitted to our hospital.

*Staphylococcus aureus* was mainly isolated from the respiratory tract (82/226), the skin and mucous membranes (64/226), and pus (47/226) (Table 5). From a total of 226 strains, 126 (55.75%) were Methicillin-resistant *Staphylococcus aureus* (MRSA) and 100 (44.25%) were Methicillin-susceptible *Staphylococcus aureus* (MSSA). The MRSA strains had the highest resistance to tetracycline (83.66%), erythromycin (83.33%), and clindamycin (73%). We did not identify any MRSA strain resistant to vancomycin, teicoplanin, linezolid, daptomycin, and quinupristin/dalfopristin. The MSSA had a resistance rate of 44.44% to tetracycline, 35.35% to erythromycin, and 31.31% to clindamycin. We did not identify any MSSA strain resistant to trimethoprim/sulfamethoxazole, vancomycin, teicoplanin, linezolid, daptomycin, quinupristin/dalfopristin, fusidic acid, and mupirocin (Figure 2).

#### 2.2.3. *Klebsiella* Species

**Figure 3:**
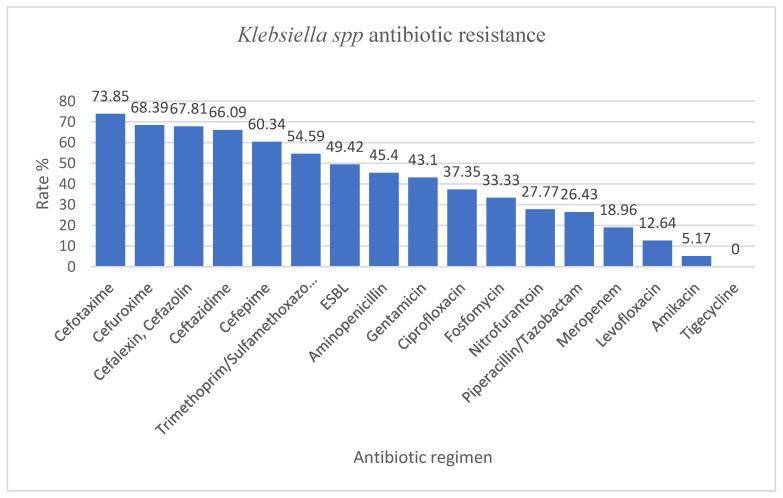
*Klebsiella* spp. antimicrobial resistance encountered in the specimens collected from the pediatric patients admitted to our hospital.

*Klebsiella* spp. included mainly *Klebsiella pneumoniae* (157/174), followed by *Klebsiella oxytoca* (12/174) and *Klebsiella ozaenae* (5/174). The strains were mainly isolated (Table 6) from the urine (41/174), the skin and mucous membranes (41/174), and the respiratory tract (30/174). *Klebsiella* spp. presented increased rates of AMR, above 50% to first, second, third, and fourth generation: cefotaxime (73.85%), cefuroxime (68.39%), cefalexin and cefazolin (67.81%), ceftazidime (66%), cefepime (60%), and also trimethoprim/sulfamethoxazole (54.59%). The highest susceptibility rates identified were to amikacin (94.8%) and tigecycline (100%) (Figure 3). The rate of MDR and XDR in our study was 43.67% and 19.54%, respectively.

#### 2.2.4. *Pseudomonas* Species

**Figure 4:**
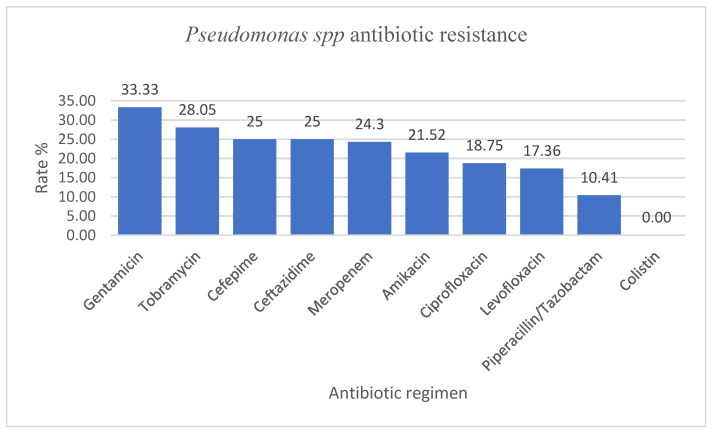
*Pseudomonas* spp. antimicrobial resistance encountered in the specimens collected from the pediatric patients admitted to our hospital.

*Pseudomonas* spp. comprising mainly *Pseudomonas aeruginosa* (137/144) were mainly isolated (Table 7) from the respiratory tract (66/144), the urine (16/144), and the skin and mucous membranes (15/144). *Pseudomonas* spp. had AMR values in the range of 10–35% to gentamicin (33.3%), tobramycin (28%), cefepime and ceftazidime (25%), meropenem (24.3%), amikacin (21.52%), ciprofloxacin (18.75%), levofloxacin (17.36%), and piperacillin/tazobactam (10.41%). Instead, all the strains of *Pseudomonas* spp. had a 100% susceptibility rate to colistin (Figure 4).

#### 2.2.5. *Enterococcus* Species

**Figure 5:**
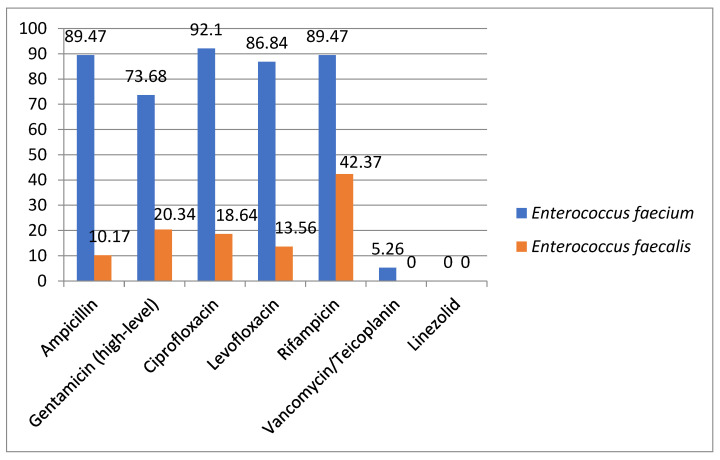
*Enterococcus faecium* and *Enterococcus faecalis* antimicrobial resistance.

*Enterococcus* spp. included *Enterococcus faecium* (38 strains), *Enterococcus faecalis* (59 strains), *Enterococcus gallinarium* (4 strains), *Enterococcus durans* (5 strains), and *Enterococcus avium* (1 strain). They were isolated mostly from the skin and mucous membranes (31/107), the urine (24/107), and the blood (15/107) (see Table 8). The resistance rates for *Enterococcus faecium* compared to *Enterococcus faecalis* are presented in Figure 5.

To summarize, the AMR rates for the top five bacteria isolated from the specimens are presented in Table 9.

## 3. Discussion

The incidence of multidrug-resistant organisms is on the rise, causing substantial morbidity and mortality among patients. Infections caused by MDR bacteria pose greater treatment challenges, leading to more severe and protracted illnesses, which in turn result in longer hospital stays. These infections have been shown to increase hospitalization times by up to 20% and to be associated with poorer outcomes, including a mortality rate that may be as high as 40% in cases of hospital-acquired MDR infections [15].

Given the rapidly evolving context, failure to take urgent action may lead us into a post-antibiotic era where common infections become fatal once again. This is particularly valid for children who are more susceptible to common infections.

Our study aimed to examine the distribution of the main pathogens and the prevalence of AMR at Marie Curie Emergency Children’s Hospital during the last year. Due to the potential serious infections occurring in hospitalized patients caused by antibiotic-resistant GN and GP bacteria, physicians are concerned with identifying the presence and spread of these agents [16,17]. Choosing and prescribing effective antibiotics for the treatment of pediatric infections presents a significant challenge. This is further complicated by the fact that certain categories of antibiotic regimens cannot be used in neonates and children and that the patterns of AMR may vary across different regions [18]. Therefore, knowing the AMR distribution could help healthcare providers to prescribe the most effective drugs [19]. Additionally, the absence of adequate surveillance for AMR could lead to the inappropriate use of antibacterial agents by healthcare providers and patients, leading to significant healthcare issues, particularly in developing countries such as Romania [20].

In our study, from 1445 isolated microorganisms, the majority were GN (64.49%), and 34.8% were GP. These results are similar to the China Antimicrobial Surveillance Network (CHINET) [8]. Among Gram-negative bacteria, *Escherichia coli* was the most frequently isolated, and among Gram-positive, *Staphylococcus aureus* was the most frequently isolated. The most frequent ten isolated bacterial species in our study are mentioned in Table 1. Except for *Escherichia coli*, which was at the top of the list, the distribution was largely different in the international reports of CHINET and China Antimicrobial Resistance Surveillance System (CARSS) in 2016 [8,21]. The reported top ten bacterial pathogens in children by the Infectious Disease Surveillance of Pediatrics (ISPED) were *Escherichia coli*, *Streptococcus pneumoniae*, *Staphylococcus aureus*, *Haemophillus influenzae*, *Klebsiella pneumoniae*, *Moraxella catarrhalis*, *Streptococcus pyogenes*, *Staphylococcus epidermidis*, *Pseudomonas aeruginosa*, and *Acinetobacter baumannii* [6].

*Escherichia coli* had the lowest resistance rates to amikacin, meropenem, and fosfomycin (0.29%, 0.29%, and 0.01%); the values were comparable with the results of a three-year study conducted in Bologna, Italy. In this study, *Escherichia coli* resistance rate to amikacin and fosfomycin was 0.6%. They also obtained a high resistance rate to amoxicillin-clavulanate and cotrimoxazole, comparable to our results. On the other hand, in this Italian study, the resistance rate to cefotaxime was 5.8%, much lower than in our study (44.59%) [22]. The ESBL-producing *Escherichia coli* resistance rate was 18.65% in our study. This is almost similar to a John Hopkins study where the ESBL-producing *Escherichia coli* resistance rate was 13.2% [23]. The risk factors associated with high ESBL levels were long hospital stays, previous use of antibiotics, and previous Intensive Care Unit (ICU) admission [24,25,26]. In our hospital, many patients with serious diseases require a wide spectrum of antimicrobial regimens and ICU passages, which could explain the higher rate of ESBL. In the John Hopkins study, the ESBL were sensitive to amikacin and carbapenems (ertapenem, meropenem), which could indicate the use of these antibiotics in patients at risk until the result of the culture is obtained [23].

*Staphylococcus aureus* strains were divided into MSSA, which represented 100/226 (44.25%), and MRSA, which represented 126/226 (55.75%). The data in our analysis are consistent with those reported by our country to the European Antibiotic Resistance Surveillance Network (EARS-Net), which shows an increased rate of MRSA isolated from invasive infections and places Romania in the undesirable leading position in this regard (47.3% of all *Staphylococcus aureus* strains reported in 2020, which is 2.84 times higher than the estimated weighted average for participating countries). In 2021, it decreased to 41%, a decrease without statistical significance [27,28]. The rate of MRSA antibiotic resistance in our hospital is higher than the rate reported in the literature. In a study conducted by La Vecchia et al. in Milan during 2017–2021, the rate of MSSA antibiotic resistance was 70%, and the rate of MRSA antibiotic resistance was only 30% [29]. Additionally, in a study from Utah, USA, by Crandall et al., the AMR for MSSA was 79%, and for MRSA, only 21% [30]. MRSA strains typically affect people who are vulnerable due to factors such as recent hospitalization or use of healthcare services, living in long-term care facilities, undergoing hemodialysis, or having percutaneous medical devices and catheters [31,32]. In our hospital, there are many hospitalized patients having these risks, but further studies are needed to be done to investigate the high prevalence of MRSA strains. According to several studies, linezolid and vancomycin were effective against *Staphylococcus aureus* strains, so no AMR for vancomycin-resistant *Staphylococcus aureus* (VRSA) strains was declared [19,29,33].

*Klebsiella* spp. isolated in our hospital presented with an increased AMR of more than 50% resistance rate to cefotaxime, cefuroxime, cefalexin, cefazolin, ceftazidime, cefepime, trimethoprim/sulfamethoxazole, values much higher than the ones reported by Ballén et al. where the highest resistance rate was to ciprofloxacin (41%). The resistance rates to third-generation cephalosporins were similar to those reported by our country to EARS-Net (around 70% in our study vs. 70.8%), as well as the resistance to aminoglycosides (43.1% in our study vs. 51.6%). However, unlike the data from EARS-Net, we found lower resistance rates to meropenem (18.69% in our study vs. 54.5%) and fluoroquinolones (more than two times less in our study, probably due to the fact that this class of antibiotics is less used in children) [27]. In that study by Ballén et al., 40.16% of the strains were considered MDR, and 1.57% were considered XDR. The rate of MDR and XDR in our study were 43.67% and 19.54%, respectively [34]. The AMR for ESBL-producing *Klebsiella* spp. was 49.42%. In our study, the highest susceptibility was for amikacin and tigecycline. *Klebsiella* spp. are identified as members of a group that includes six highly virulent and antibiotic-resistant bacterial pathogens: *Enterococcus faecium*, *Staphylococcus aureus*, *Klebsiella pneumoniae*, *Acinetobacter baumannii*, *Pseudomonas aeruginosa*, and *Enterobacter* spp. (ESKAPE group). This group is known for its characteristic ability to resist or avoid the action of antimicrobial agents [35]. In addition, the World Health Organization has designated *Klebsiella pneumoniae* as a high-priority species and advocates for the development of new antibiotics due to the growing global issue of antimicrobial resistance [36].

*Pseudomonas* spp. had resistance rate values to ceftazidime, meropenem, amikacin, ciprofloxacin, and piperacillin/tazobactam ranging from 10 to 35%, while the Infectious Disease Surveillance of Pediatrics (ISPED) program reported values of 2.3–10.1% [6]. However, compared to the data reported to EARS-Net by our country, in our study, we identified lower rates of resistance to piperacillin-tazobactam (10.41% vs. 47.2%), ceftazidime (25% vs. 46%), meropenem (24.3% vs. 45.9%), ciprofloxacin/levofloxacin (18.75% and 17.36% vs. 45.7%), and gentamycin (33.33% vs. 41.7%) [27].

*Enterococcus* spp. had the highest antimicrobial resistance rates, as expected, among *Enterococcus faecium* strains compared to *Enterococcus faecalis*. Similar levels of resistance were seen in the results reported by ISPED [6]. We also found two isolates of *Enterococcus facecium* resistant to vancomycin and teicoplanin. This resistance profile is of concern for our country, as between 2012 and 2020, glycopeptide resistance increased significantly, from 2.9% to 41.7%; the level of resistance is now the fourth highest among EU/EEA countries [28].

Overall, our results reveal a higher antimicrobial resistance to the main pathogens when compared to the literature findings [37]. However, we need to mention that our hospital is classified as a tertiary care center, providing advanced and specialized medical care to patients from all over the country who suffer from complex medical conditions or illnesses. With a broad range of medical specialties, including surgical, medical, and intensive care, our center offers specialized services and complex procedures that are not commonly found in general hospitals or clinics. The high prevalence of intricate diseases among our patient population, necessitating prolonged hospital stays and the administration of multiple antibiotic regimens has been identified as a significant contributing factor to the rise in AMR rates. This concerning trend poses serious challenges in terms of available antibiotic treatment options, greatly limiting their effectiveness and often necessitating the use of reserve or watchlist antibiotics. Furthermore, the hospital environment provides an ideal breeding ground for the transmission and spread of antibiotic-resistant germs, leading to the emergence of difficult-to-control outbreaks of healthcare-associated infections. Last but not least, AMR has an economic impact by increasing treatment costs and prolonging hospitalization.

In June 2018, European Center for Disease Control (ECDC) evaluated the AMR levels in Romania. It was considered that our country presents with a high AMR compared to other states of Europe. There are several factors likely to contribute to this situation, such as excessive use of antibiotics in the general population, excessive use of last-line broad-spectrum antibiotics in hospitals, inadequate local AMR surveillance, which could contribute to proper use of antimicrobial agents, and scarce antimicrobial stewardship antimicrobial programs [38]. Recently, awareness campaigns have been organized to limit the use of antibiotics and the sale of over-the-counter antimicrobial agents, but there are still many efforts to be made.

### Study Limitations

This study has several limitations that need to be addressed. Firstly, the analysis period was relatively short, and it is crucial to continuously monitor and analyze the emerging trends of antibiotic resistance. This requires the implementation of effective antimicrobial stewardship programs, as the misuse and overuse of antibiotics contribute significantly to the increasing antimicrobial resistance. Additionally, this study was conducted in a single pediatric hospital in Bucharest, which may limit the generalizability of the findings. Conducting a multicenter study, including territorial hospitals, over a more extended period and including demographic data could provide more accurate data on the antimicrobial resistance issue in Romania. Furthermore, it is important to note that our study was conducted within a specific timeframe, namely during the COVID-19 pandemic. This unique and challenging period may have influenced our results in various ways. Additionally, during the study, we identified a significant problem with the outsourced microbiology laboratory in our hospital and the lack of a clinical microbiology specialist. This situation hinders good communication between physicians and the laboratory when unusual resistance profiles are observed. In such situations, we had to send samples to a national reference institute for confirmation, which is a time-consuming and costly process. The lack of effective communication may lead to the overlooking of critical situations that need to be clarified, ultimately impacting both the analysis of resistance data and the clinical management of the patient. Therefore, it is crucial to have a clinical microbiology specialist in our hospital to facilitate effective communication and proper management of patients with antimicrobial resistance issues.

## 4. Materials and Methods

We conducted a retrospective analysis of clinical specimens obtained from patients admitted to the Marie Curie Emergency Children’s Hospital between 1 January 2022 and 31 December 2022. The study included a total of 1445 clinical isolates, consecutive and non-duplicated, collected from hospitalized patients from various departments (ICU, NICU, cardiovascular ICU, cardiology, oncology, orthopedics, ENT, pediatrics, nephrology, hemodialysis, and neurosurgery).

The samples were collected for etiological identification in patients with suggestive symptomatology of acute infection. In our hospital exists portage identification protocols, notably in patients who will undergo surgery (orthopedics, cardiology, pediatric surgery) and in high-risk patients (ICU, NICU, oncology) for whom samples were taken.

Microbiological samples were processed in the outsourced microbiology laboratory, as the hospital did not have its own laboratory at the time of analysis. Each specimen was inoculated on a different medium according to the manufacturer and incubated at 37 °C for 24 h. The urine cultures were inoculated on CHROMagar medium, the nasal and pharyngeal samples on Columbia and Chocolat medium (Biomérieux), and stool cultures on Selenit enrichment broth (Sanimed) and Hektoen agar (Biomérieux). The other specimens (blood, cerebrospinal fluid—CSF, sputum, pus, auricular, ocular, vagina, urethral) were inoculated on several mediums, namely Columbia, Chocolat, MacConkey, and Brain heart infusion broth (Table 10). Various kits were used for the identification of certain bacteria—Staphytect Plus (Oxoid) for coagulase-positive *Staphylococci*, Streptococcal grouping kit (Oxoid) for hemolytic *Streptococci* and API NH kit (Biomérieux) for the identification of *Neisseria, Haemophilus,* and *Moraxella catharralis* (Table 11). Identification and antimicrobial resistance were performed by the MicroScan Automated System (Beckman Coulter). For identification and AMR testing, we used 3 types of standardized panel kits: one for Gram-negative bacteria, one panel for Gram-positive bacteria, and MICroSTREP plus 1 for streptococci. Each kit tested for certain antibiotics (Table 12), and the results were reported depending on the specimen type. The AMR was interpreted in accordance with the Clinical and Laboratory Standards Institute (CLSI) guidelines. Extended-spectrum beta-lactamase-producing (ESBL)-producing bacteria were also identified automatically by MicroScan System.

We excluded bacterial duplicates, defined as the same germ with the same resistance profile isolated from the same patient within less than four weeks. We also excluded from the analysis antibiotics for which too few strains were tested to be relevant (below 30).

An isolated microorganism was classified as MDR if it demonstrated in vitro non-susceptibility to one or more agents within three or more antimicrobial categories. If a bacterium was resistant to at least one agent in almost all antimicrobial categories, except two or less, it was classified as extensively drug-resistant (XDR). Finally, the isolate was classified as pan-drug-resistant (PDR) if it was non-susceptible to all the listed antimicrobial agents [34].

## 5. Conclusions

The results of our study identified an increased AMR rate to commonly used antibiotics in pediatric patients from our hospital. In a country with excessive use of antibiotics, these results reinforce the ECDC’s signal alarm regarding the problem of antimicrobial resistance. We consider it important to continue the monitoring and research of antibiotic resistance in pediatric hospitals due to its economic and therapeutic implications.

## Figures and Tables

**Table 1:** Distribution of the main isolated pathogens.

Bacterial Species	Number of Isolates	Percent
*Escherichia coli*	343	23.73%
*Staphylococcus aureus*	226	15.64%
*Klebsiella* spp.	174	12.04%
*Pseudomonas* spp.	144	9.96%
*Coagulase*-negative Staphylococci *	128	8.85%
*Enterococcus* spp.	107	7.4%
Other non-fermentative Gram-negative bacilli **	64	4.42%
*Acinetobacter* spp.	51	3.52%
Other Gram-negative ***	47	3.25%
*Proteus* spp.	42	2.9%
*Enterobacter* spp.	41	2.83%
*Streptococcus pneumoniae*	15	1.03%
*Salmonella* spp.	14	0.96%
Other *Streptococcus*	14	0.96%
*Beta hemolytic Streptococcus*	13	0.89%
*Serratia marcescens*	10	0.69%
*Candida*	10	0.69%
*Haemophilus influenzae*	2	0.13%
Total	1445	100%

* Coagulase-negative *Staphylococci*: *Staphylococcus epidermidis*, *Staphylococcus hominis*, *Staphylococcus haemolyticus*, *Staphylococcus simulans*, *Staphylococcus warnerii*, *Staphylococcus schleiferi*, *Staphylococcus auricularis*, *Staphylococcus capitis*. ** *Stenotrophomonas maltophilia* *** Other Gram-negative: *Citrobacter braaki*, *Citrobacter murliniae*, *Citrobacter freundii*, *Citrobacter koseri*, *Citrobacter sedlakii*, *Emperobacter brevis*, *Kluyvera intermedia*, *Morganella morganii*, *Pantoea agglomerans*, *Providencia rustigianii*, *Providencia stuartii*, *Raoutella ornitholytica*, *Shigella sonnei*, *Vibrio alginolyticus*, *Yersinia ruckeri*.

**Table 2:** Distribution of the isolated specimen types and top five pathogens.

Isolates	No. (Percent)	Species				
		Pathogen 1	Pathogen 2	Pathogen 3	Pathogen 4	Pathogen 5
Urine	331 (22.9%)	*Escherichia coli* (192)	*Klebsiella* spp. (41)	*Enterococcus* (24)	*Proteus* spp. (21)	*Pseudomonas* spp. (16)
Respiratory tract	262 (18.13%)	*Staphylococcus aureus* (82)	*Pseudomonas* spp. (66)	*Klebsiella* spp. (30)	Other Gram-negative (23)	*Enterobacter* (13)
Skin/nose/pharynx	249 (17.23%)	*Staphylococcus aureus* (64)	*Klebsiella* spp. (41)	*Enterococcus* (31)	*Escherichia coli* (20)	*Pseudomonas* spp. (15)
Blood	182 (12.59%)	*Coagulase-negative Staphylococci* (72)	*Klebsiella* spp. (24)	*Other Gram-negative* (18)	*Enterococcus* (15)	*Pseudomonas* spp. (13)
Pus	114 (7.88%)	*Staphylococcus aureus* (47)	*Escherichia coli* (18)	*Coagulase-negative Staphylococci (13)*	*Enterococcus* (9)	*Pseudomonas* spp. (9)
Central venous catheter	87 (6.02%)	*Coagulase*-negative *Staphylococci* (20)	*Enterococcus* (10)	*Pseudomonas* spp. (7)	*Klebsiella* spp. (7)	*Escherichia coli* (7)
Peritoneal fluid	86 (5.95%)	*Escherichia coli* (33)	*Pseudomonas* spp. (11)	*Enterococcus* (9)	*Acinetobacter* spp. (7)	*Staphylococcus aureus* (5)
Stool	48 (3.32%)	*Escherichia coli* (34)	*Salmonella* spp. (13)	*Shigella sonnei* (1)		
Others	52 (3.59%)	*Staphylococcus aureus* (13)	*Pseudomonas* spp. (4)	*Coagulase-negative Staphylococci* (3)	*Proteus* spp. (3)	*Escherichia coli* (2)
Vaginal/urethral specimen	22 (1.52%)	*Escherichia coli* (9)	*Proteus* spp. (6)	*Staphylococcus aureus* (2)	*Klebsiella* spp. (2)	*Pseudomonas* spp. (1)
Cerebrospinal fluid	12 (0.83%)	*Enterococcus* (4)	*Escherichia coli* (3)	*Pseudomonas* spp. (2)	*Coagulase*-negative *Staphylococci* (2)	*Staphylococcus aureus* (1)
Total	1445					

**Table 3:** Distribution of the pathogens per department.

Department/Bacterial Species	ICU	Cardiovascular ICU	NICU	Neurosurgery	Cardiology	Orthopedics	ENT	Pediatrics	Nephrology	Surgery	Oncology	Hemodialysis	Emergency Department	Total
*Escherichia coli*	10	6	23	5	12	2	1	95	92	92	3	0	2	343
*Klebsiella* spp.	27	11	31	3	13	0	2	35	32	20	0	0	0	174
*Pseudomonas* spp.	17	5	22	3	6	1	5	56	11	15	3	0	0	144
*Acinetobacter* spp.	8	5	5	0	9	0	0	5	11	0	8	0	0	51
*Other non-fermentative Gram-negative bacilli*	4	9	16	0	3	0	1	16	14	1	0	0	0	64
*Proteus*	2	1	6	0	2	0	0	4	17	10	0	0	0	42
*Serratia*	3	0	2	0	0	0	0	4	0	0	1	0	0	10
*Enterobacter*	6	6	8	0	3	0	0	10	4	4	0	0	0	41
*Salmonella*	0	0	1	0	0	0	0	12	1	0	0	0	0	14
*Other Gram-negative*	4	0	7	0	7	0	0	11	12	4	2	0	0	47
*Staphylococcus aureus*	22	13	22	2	28	10	8	80	13	23	1	4	0	226
*Coagulase-negative Staphylococci*	4	16	20	4	7	2	0	42	15	10	8	0	0	128
*Enterococcus*	4	3	19	4	21	2	0	15	17	20	2	0	0	107
*Other cocci*	3	1	2	1	3	1	5	11	8	6	1	0	0	42
*Hemophilus influenzae*	0	0	0	0	1	0	0	1	0	0	0	0	0	2
*Candida*	1	1	2	0	0	0	0	2	3	1	0	0	0	10

**Table 4:** The specimen types from which *Escherichia coli* was isolated.

Specimen Type	Number of Isolates	Percent
Urine	192	55.97%
Stool	34	9.91%
Peritoneal fluid	33	9.62%
Skin/nose/pharynx	31	9.03%
Pus	18	5.24%
Respiratory tract	9	2.62%
Vaginal/urethral specimens	9	2.62%
Central venous catheter	7	2.04%
Blood	5	1.45%
Cerebrospinal fluid	3	0.87%
Others	2	0.58%
Total	343	100%

**Table 5:** The specimen types from which *Staphylococcus aureus* was isolated.

Specimen Type	Number of Isolates	Percent
Respiratory tract	82	36.28%
Skin/nose/pharynx	64	28.31%
Pus	47	20.79%
Others	7	3.09%
Blood	6	2.65%
Peritoneal fluid	5	2.21%
Central venous catheter	5	2.21%
Ocular specimen	4	1.76%
Vaginal/urethral specimens	2	0.88%
Auricular specimens	2	0.88%
Urine	1	0.44%
Cerebrospinal fluid	1	0.44%
Total	226	100%

**Table 6:** Specimen types from which *Klebsiella* spp. were isolated.

Specimen Type	Number of Isolates	Percent
Urine	41	23.56%
Skin/nose/pharynx	41	23.56%
Respiratory tract	30	17.24%
Blood	24	13.79%
Central venous catheter	21	12.06%
Pus	8	4.59%
Peritoneal fluid	7	4.02%
Vaginal/urethral specimens	1	0.57%
Others	1	0.57%
Total	174	100%

**Table 7:** Specimen types from which *Pseudomonas* spp. were isolated.

Bacterial Species	Number of Isolates	Percent
Respiratory tract	66	45.83%
Urine	16	11.11%
Skin/nose/pharynx	15	10.41%
Blood	13	9.02%
Peritoneal fluid	11	7.63%
Pus	9	6.25%
Central venous catheter	7	4.86%
Others	4	2.77%
Cerebrospinal fluid	2	1.38%
Vaginal/urethral specimens	1	0.69%
Total	144	100%

**Table 8:** Specimen types from which *Enterococcus* spp. were isolated.

Bacterial Species	Number of Isolates	Percent
Skin/nose/pharynx	31	28.97%
Urine	24	22.42%
Blood	15	14.01%
Central venous catheter	10	9.34%
Peritoneal fluid	9	8.41%
Pus	9	8.41%
Respiratory tract	5	4.67%
Cerebrospinal fluid	4	3.73%
Total	107	100%

**Table 9:** The pattern of AMR in the top five bacteria isolated.

	*Escherichia coli*	MRSA	MSSA	*Klebsiella* spp.	*Pseudomonas* spp.	*Enterococcus* spp.
Amikacin	1/343 (0.29%)			9/174 (5.17%)	31/144 (21.52%)	
Aminopenicillin	49/343 14.28%)			79/174 (45.4%)		
Ampicillin	232/343 (67.63%)					43/106 (40.56%)
Cefalexin, Cefazolin	74/343 (21.57%)			118/174 (67.81%)		
Cefepime	68/343 (19.82%)			105/174 (60.34%)	36/144 (25%)	
Cefotaxime	33/74 (44.59%)			113/153 (73.85%)		
Ceftazidime	69/343 (20.11%)			115/174 (66.09%)	36/144 (25%)	
Cefuroxime	71/343 (20.69%)			119/174 (68.39%)		
Ciprofloxacin	60/343 (17.49%)	17/126 (13.49%)	5/100 (5%)	65/174 (37.35%)	27/144 (18.75%)	48/106 (45.28%)
Clindamycin		92/126 (73.01%)	31/99 (31.31%)			
Colistin					0/143 (0%)	
Daptomycin		0/80 (0%)	0/100 (0%)			
Erythromycin		105/126 (83.33%)	35/99 (35.35%)			
ESBL	64/343 (18.65%)			86/174 (49.42%)		
Fosfomycin	2/200 (0.01%)			2/6 (33.33%)		
Fusidic acid		2/126 (1.58%)	0/100 (0%)			
Gentamicin	25/343 (7.28%)	26/126 (20.63%)	11/100 (11%)	75/174 (43.1%)	48/144 (33.33%)	41/106 (38.67%)
Levofloxacin	54/343 (15.74%)	16/126 (12.69%)	5/100 (5%)	22/174 (12.64%)	25/144 (17.36%)	43/106 (40.56%)
Linezolid		0/103 (0%)	0/100 (0%)			0/106 (0%)
Meropenem	1/343 (0.29%)			33/174 (18.96%)	35/144 (24.3%)	
Moxifloxacin		14/78 (17.94%)	5/100 (5%)			
Mupirocin		1/125 (0.8%)	0/100 (0%)			
Nitrofurantoin	3/179 (1.67%)			5/18 (27.77%)		1/24 (4.16%)
Oxacillin		126/226 (55.75%)				
Piperacillin/Tazobactam	7/343 (2.04%)			46/174 (26.43%)	15/144 (10.41%)	
Quinupristin/dalfopristin		0/103 (0%)	0/100 (0%)			
Rifampin		7/126 (5.55%)	1/100 (1%)			56/106 (52.83%)
Teicoplanin		0/103 (0%)	0/100 (0%)			0/106 (0%)
Tetracycline		41/49 (83.67%)	16/36 (44.44%)			
Tigecycline				0/174 (0%)		
Tobramycin					39/139 (28.05%)	
Trimethoprim/Sulfamethoxazole	140/343 (40.81%)	1/126 (0.79%)	0/100 (0%)	95/174 (54.59%)		
Vancomycin		0/103 (0%)	0/100 (0%)			4/106 (3.77%)

**Table 10:** Medium samples.

Sample	Medium	Enrichment Medium
Urine	CHROMagar	
Nasal/pharyngeal	Columbia, Chocolat	
Stool	Hektoen agar	Selenit enrichment broth
Others	Columbia, Chocolat, MacConkey	Brain heart infusion broth

**Table 11:** Panel kits.

Bacteria	Kit
*Coagulase*-positive *Staphylococci*	Staphytect Plus
Hemolytic *Streptococci*	Streptococcal grouping kit
*Neisseria*. *Hemophilus*, *Moraxella*	API NH kit

**Table 12:** Antibiotic testing kits.

Gram-Negative Bacteria	Gram-Positive Bacteria	*Streptococci*
Amikacin		
Amoxicillin/Clavulanic acid	Amoxicillin/Clavulanic acid	Amoxicillin/Clavulanic acid
Ampicillin	Ampicillin	Ampicillin
Aztreonam	Cefoxitine	Azithromycin
Cefepime	Cephalothin	Cefaclor
Cefotaxime	Chloramphenicol	Cefepime
Cefotaxime/Clavulanic acid	Ciprofloxacin	Cefotaxime
Cefoxitine	Clarithromycin	Ceftriaxone
Ceftazidime	Clindamycin	Cefuroxime
Cefuroxime	Daptomycin	Chloramphenicol
Chloramphenicol	Erythromycin	Clindamycin
Ciprofloxacin	Fosfomycin	Erythromycin
Colistin	Fusidic acid	Levofloxacin
Ertapenem	Gentamicin	Meropenem
Fosfomycin	Inducible Clindamycin	Penicillin
Gentamicin	Levofloxacin	Tetracycline
Imipenem	Linezolid	Trimethroprime/Sulfametoxazole
Levofloxacin	Moxifloxacin	Vancomycin
Mecillinam	Netilmicin	
Meropenem	Nitrofurantoin	
Nalidixic acid	Norfloxacin	
Nitrofurantoin	Oxacillin	
Piperacillin/Tazobactam	Penicillin	
Piperacillin	Rifampin	
Tetracycline	Synercid	
Tigecycline	Teicoplanin	
Tobramycin	Tetracycline	
Trimethroprime/Sulfametoxazole	Trimethroprime/Sulfametoxazole	
Trimethoprim	Vancomycin	

## Data Availability

Data sharing is not applicable to this article.

## References

[1] Gandra S., Alvarez-Uria G., Turner P., Joshi J., Limmathurotsakul D., van Doorn H.R. (2020). Antimicrobial Resistance Surveillance in Low- and Middle-Income Countries: Progress and Challenges in Eight South Asian and Southeast Asian Countries. Clin. Microbiol. Rev..

[2] Huemer M., Shambat S.M., Brugger S.D., Zinkernagel A.S. (2020). Antibiotic resistance and persistence—Implications for human health and treatment perspectives. EMBO Rep..

[3] Le Doare K., Bielicki J., Heath P.T., Sharland M. (2014). Systematic Review of Antibiotic Resistance Rates Among Gram-Negative Bacteria in Children with Sepsis in Resource-Limited Countries. J. Pediatr. Infect. Dis. Soc..

[4] Diallo O.O., Baron S.A., Abat C., Colson P., Chaudet H., Rolain J.-M. (2020). Antibiotic resistance surveillance systems: A review. J. Glob. Antimicrob. Resist..

[5] Dever L.A., Dermody T.S. (1991). Mechanisms of bacterial resistance to antibiotics. Arch. Intern. Med..

[6] Fu P., Xu H., Jing C., Deng J., Wang H., Hua C., Chen Y., Chen X., Zhang T., Zhang H. (2021). Bacterial Epidemiology and Antimicrobial Resistance Profiles in Children Reported by the ISPED Program in China, 2016 to 2020. Microbiol. Spectr..

[7] Tanwar J., Das S., Fatima Z., Hameed S. (2014). Multidrug Resistance: An Emerging Crisis. Interdiscip. Perspect. Infect. Dis..

[8] Hu F., Zhu D., Wang F., Wang M. (2018). Current Status and Trends of Antibacterial Resistance in China. Clin. Infect. Dis..

[9] Veeraraghavan B., Walia K. (2019). Antimicrobial susceptibility profile & resistance mechanisms of Global Antimicrobial Resistance Surveillance System (GLASS) priority pathogens from India. Indian J. Med Res..

[10] Martinez-Vega R., Jauneikaite E., Thoon K.C., Chua H.Y., Chua A.H., Khong W.X., Tan B.H., Hong J.L.G., Venkatachalam I., Tambyah P.A. (2019). Risk factor profiles and clinical outcomes for children and adults with pneumococcal infections in Singapore: A need to expand vaccination policy?. PLoS ONE.

[11] Campbell A.J., Daley D.A., Bell J.M., Pang S., Coombs G.W., Carapetis J.R., Bowen A.C., Blyth C.C. (2020). Progress towards a coordinated, national paediatric antimicrobial resistance surveillance programme: Staphylococcus aureus, enterococcal and Gram-negative bacteraemia in Australia. J. Antimicrob. Chemother..

[12] Aslam B., Wang W., Arshad M.I., Khurshid M., Muzammil S., Nisar M.A., Alvi R.F., Aslam M.A., Qamar M.U., Salamat M.K.F. (2018). Antibiotic resistance: A rundown of a global crisis. Infect. Drug Resist..

[13] Tsutsui A., Suzuki S. (2018). Japan nosocomial infections surveillance (JANIS): A model of sustainable national antimicrobial resistance surveillance based on hospital diagnostic microbiology laboratories. BMC Heal. Serv. Res..

[14] Gandra S., Mojica N., Klein E.Y., Ashok A., Nerurkar V., Kumari M., Ramesh U., Dey S., Vadwai V., Das B.R. (2016). Trends in antibiotic resistance among major bacterial pathogens isolated from blood cultures tested at a large private laboratory network in India, 2008–2014. Int. J. Infect. Dis..

[15] Romandini A., Pani A., Schenardi P.A., Pattarino G.A.C., De Giacomo C., Scaglione F. (2021). Antibiotic Resistance in Pediatric Infections: Global Emerging Threats, Predicting the Near Future. Antibiotics.

[16] Folgori L., Bielicki J., Heath P., Sharland M. (2017). Antimicrobial-resistant Gram-negative infections in neonates. Curr. Opin. Infect. Dis..

[17] Jorak A., Keihanian F., Saeidinia A., Heidarzadeh A., Saeidinia F. (2014). A Cross Sectional Study on Knowledge, Attitude and Practice of Medical Students Toward Antibiotic Resistance and its Prescription, Iran. Adv. Environ. Biol..

[18] Keihanian F., Saeidinia A., Abbasi K., Keihanian F. (2018). Epidemiology of antibiotic resistance of blood culture in educational hospitals in Rasht, North of Iran. Infect. Drug Resist..

[19] Azimi T., Maham S., Fallah F., Azimi L., Gholinejad Z. (2019). Evaluating the antimicrobial resistance patterns among major bacterial pathogens isolated from clinical specimens taken from patients in Mofid Children’s Hospital, Tehran, Iran: 2013–2018. Infect. Drug Resist..

[20] Prestinaci F., Pezzotti P., Pantosti A. (2015). Antimicrobial resistance: A global multifaceted phenomenon. Pathog. Glob. Health.

[21] Rețeaua națională de supraveghere a rezistenței bacteriene—Acasă. http://www.carss.cn/.

[22] Pierantoni L., Andreozzi L., Ambretti S., Dondi A., Biagi C., Baccelli F., Lanari M. (2021). Three-Year Trend in Escherichia coli Antimicrobial Resistance among Children’s Urine Cultures in an Italian Metropolitan Area. Children.

[23] Degnan L.A., Milstone A.M., Diener-West M., Lee C.K.K. (2015). Extended-Spectrum Beta-Lactamase Bacteria From Urine Isolates in Children. J. Pediatr. Pharmacol. Ther..

[24] Arnoni M.V., Berezin E.N., Martino M.D. (2007). Risk factors for nosocomial bloodstream infection caused by multidrug resistant gram-negative bacilli in pediatrics. Braz. J. Infect. Dis..

[25] Kim Y.-K., Pai H., Lee H.-J., Park S.-E., Choi E.-H., Kim J., Kim J.-H., Kim E.-C. (2002). Bloodstream Infections by Extended-Spectrum β-Lactamase-Producing *Escherichia coli* and *Klebsiella pneumoniae* in Children: Epidemiology and Clinical Outcome. Antimicrob. Agents Chemother..

[26] Zaoutis T.E., Goyal M., Chu J.H., Coffin S.E., Bell L.M., Nachamkin I., McGowan K.L., Bilker W.B., Lautenbach E. (2005). Risk Factors for and Outcomes of Bloodstream Infection Caused by Extended-Spectrum β-Lactamase–Producing *Escherichia coli* and *Klebsiella* Species in Children. Pediatrics.

[27] Consumul-de-antibiotice-Rezistenta-Microbiana-si-Infectii-Asociate-Asistentei-Medicale-in-Romania-2020—Institutul Național de Sănătate Publică, 30 September 2022. https://insp.gov.ro/wpfb-file/consumul-de-antibiotice-rezistenta-microbiana-si-infectii-asociate-asistentei-medicale-in-romania-2020-pdf/.

[28] European Centre for Disease Prevention and Control and World Health Organization (2022). Antimicrobial Resistance Surveillance in Europe: 2022: 2020 Data.

[29] La Vecchia A., Ippolito G., Taccani V., Gatti E., Bono P., Bettocchi S., Pinzani R., Tagliabue C., Bosis S., Marchisio P. (2022). Epidemiology and antimicrobial susceptibility of Staphylococcus aureus in children in a tertiary care pediatric hospital in Milan, Italy, 2017—2021. Ital. J. Pediatr..

[30] Crandall H., Kapusta A., Killpack J., Heyrend C., Nilsson K., Dickey M., Daly J.A., Ampofo K., Pavia A.T., Mulvey M.A. (2020). Clinical and molecular epidemiology of invasive Staphylococcus aureus infection in Utah children; continued dominance of MSSA over MRSA. PLoS ONE.

[31] Lesens O., Hansmann Y., Brannigan E., Hopkins S., Meyer P., O’Connel B., Prévost G., Bergin C., Christmann D. (2005). Healthcare-Associated *Staphylococcus aureus* Bacteremia and the Risk for Methicillin Resistance: Is the Centers for Disease Control and Prevention Definition for Community-Acquired Bacteremia Still Appropriate?. Infect. Control. Hosp. Epidemiol..

[32] Fernando S.A., Gray T.J., Gottlieb T. (2017). Healthcare-acquired infections: Prevention strategies. Intern. Med. J..

[33] Dharmapalan D., Shet A., Yewale V., Sharland M. (2017). High Reported Rates of Antimicrobial Resistance in Indian Neonatal and Pediatric Blood Stream Infections. J. Pediatr. Infect. Dis. Soc..

[34] Ballén V., Gabasa Y., Ratia C., Ortega R., Tejero M., Soto S. (2021). Antibiotic Resistance and Virulence Profiles of Klebsiella pneumoniae Strains Isolated from Different Clinical Sources. Front. Cell. Infect. Microbiol..

[35] Santajit S., Indrawattana N. (2016). Mechanisms of Antimicrobial Resistance in ESKAPE Pathogens. BioMed Res. Int..

[36] Tacconelli E. (2017). Global Priority List of Antibiotic-Resistant Bacteria to Guide Research, Discovery, and Development. https://policycommons.net/artifacts/1818147/global-priority-list-of-antibiotic-resistant-bacteria-to-guide-research-discovery-and-development/2555608/.

[37] Galoș F., Boboc C., Ieșanu M.-I., Anghel M., Ioan A., Iana E., Coșoreanu M.T., Boboc A.A. (2023). Antibiotic Resistance and Therapeutic Efficacy of *Helicobacter pylori* Infection in Pediatric Patients—A Tertiary Center Experience. Antibiotics.

[38] (2018). ECDC Country Visit to Romania to Discuss Antimicrobial Resistance Issues. https://www.ecdc.europa.eu/en/publications-data/ecdc-country-visit-romania-discuss-antimicrobial-resistance-issues.

